# mTOR inhibition as an adjuvant therapy in a metastatic model of HPV+ HNSCC

**DOI:** 10.18632/oncotarget.8286

**Published:** 2016-03-23

**Authors:** Joseph D. Coppock, Paola D. Vermeer, Daniel W. Vermeer, Kimberly M. Lee, W. Keith Miskimins, William C. Spanos, John H. Lee

**Affiliations:** ^1^ Cancer Biology Research Center, Sanford Research/USD, Sioux Falls, SD 57104, USA; ^2^ Department of Otolaryngology/Head and Neck Surgery, Sanford Health, Sioux Falls, SD 57105, USA

**Keywords:** head and neck oral cancer, human papillomavirus, metastasis, rapamycin, mTOR

## Abstract

Effective treatments for recurrent/metastatic human papillomavirus-positive (HPV+) head and neck squamous cell cancer (HNSCC) are limited. To aid treatment development, we characterized a novel murine model of recurrent/metastatic HPV+ HNSCC. Further analysis of the parental tumor cell line and its four recurrent/metastatic derivatives led to preclinical testing of an effective treatment option for this otherwise fatal disease. Reverse phase protein arrays identified key signaling cascades in the parental and recurrent/metastatic cell lines. While protein expression profiles differed among the recurrent/metastatic cell lines, activated proteins associated with the mTOR signaling cascade were a commonality. Based on these data, mTOR inhibition was evaluated as an adjuvant treatment for recurrent/metastatic disease. mTOR activity and treatment response were assessed *in vitro* by western blot, Seahorse, proliferation, clonogenic, and migration assays. Standard-of-care cisplatin/radiation therapy (CRT) versus CRT/rapamycin were compared *in vivo*. Low-dose rapamycin inhibited mTOR signaling, decreasing proliferation (43%) and migration (62%) while it enhanced CRT-induced cytotoxicity (3.3 fold) in clonogenic assays. Furthermore, rapamycin re-sensitized CRT-resistant, metastatic tumors to treatment *in vivo*, improving long-term cures (0–30% improved to 78–100%, depending on the recurrent/metastatic cell line) and limiting lymph node metastasis (32%) and lung metastatic burden (30 fold). Studies using immune compromised mice suggested rapamycin's effect on metastasis is independent of the adaptive immune response. These data suggest a role of mTOR activation in HPV+ HNSCC recurrent/metastatic disease and that adjuvant mTOR inhibition may enhance treatment of resistant, metastatic cell populations at the primary site and limit distant metastasis.

## INTRODUCTION

Nearly 600,000 cases of head and neck squamous cell cancer (HNSCC) are diagnosed annually worldwide, accounting for approximately five percent of all cancers reported in the United States alone [1, 2]. Despite the advancement of targeted and immune therapies, survival rates remain poor at only 50% [3]. The natural disease course includes local recurrence and regional lymph node metastasis; spread to cervical lymph nodes is perhaps the most important indicator of poor prognosis, decreasing overall survival by nearly 50% [3]. Though uncommon, the lungs are the most frequent site of distant spread [4]. Failure to control loco-regional disease is the leading cause of death in HNSCC [5]; patients suffer rapid clinical deterioration with typically incurable disease, as effective treatment options for this stage disease are currently extremely limited [4].

Though the incidence of head and neck cancer has decreased with smoking prevalence [6], the distinct clinical subtype that is HPV-related is increasing rapidly [7, 8]. At least 25% of HNSCCs are HPV type-16 (HPV-16) positive (HPV+), this value increasing to 60– 80% when considering oropharyngeal cancers specifically (OPSCC). HPV+ cancers typically present at more advanced stages (III-IV), with nodal involvement, and in younger patients compared to HPV-negative (HPV-) cancers, which are more often associated with smoking, drinking, and older patients [9]. Despite their more advanced stage at presentation, HPV+ OPSCCs have a clear survival benefit over HPV-regardless of treatment strategy [6]. Yet, approximately 10% of patients suffer metastasis, culminating in incurable disease [4]. The overwhelming, increasing numbers of HPV+ OPSCC cases necessitate better treatments that address specific areas where our standard-of-care treatments are failing. However, lack of an HPV+ OPSCC metastatic model recapitulating human disease currently limits study of this phenomenon.

Our laboratory previously described a murine model of HPV+ OPSCC where mouse oropharyngeal epithelial cells (MOEs) stably expressing HPV-16 E6 and E7 together with H-Ras and luciferase (mEERL cells) were generated [10, 11]. In a study using this model, lung metastases were noted in an animal that failed standard-of-care cisplatin/radiation therapy (CRT). Several clonal metastatic cell populations were identified, cultured, and confirmed to have arisen from the primary tumor. These metastatic clones were CRT-resistant, more aggressive *in vivo*, and re-metastasized to lungs at higher rates than their parental cell line, closely mimicking human disease (see companion manuscript by Vermeer *et al*). Metastatic heterogeneity was observed in these recurrent/metastatic mEERL lung metastasizing cell lines (MLMs), suggesting treatment of metastasis must not only address resistance to standard therapies but target a pathway common to a heterogeneous population of metastatic clones. Reverse phase protein array (RPPA) analysis showed that their most notable and consistently activated pathway was the mammalian (or mechanistic) target of rapamycin (mTOR) pathway. Emerging basic, preclinical, and clinical data support the importance of mTOR signaling in HNSCC progression. Dysregulated in more than 80% of HNSCCs [3], mTOR activation is an independent predictor of recurrence [12]. Using the mEERL system, we recently reported the ability of the mTOR inhibitor, rapamycin (sirolimus), to enhance CRT-induced cytotoxicity and attenuate tumor metabolism, contributing to improved immune-mediated clearance of HPV+ OPSCC [13]. Rapamycin and analogs are well-tolerated drugs with known safety and side effect profiles, having FDA approved indications including transplant and renal cell carcinoma. Here, we describe that rapamycin not only enhances treatment of multiple unique, clonal, resistant metastatic cell populations at the primary tumor site, but potentiates effects of systemic platinum-based chemotherapy and limits distant metastasis.

## RESULTS

### mTOR signaling in HPV+ OPSCC recurrent/metastatic disease

**Figure 1:**
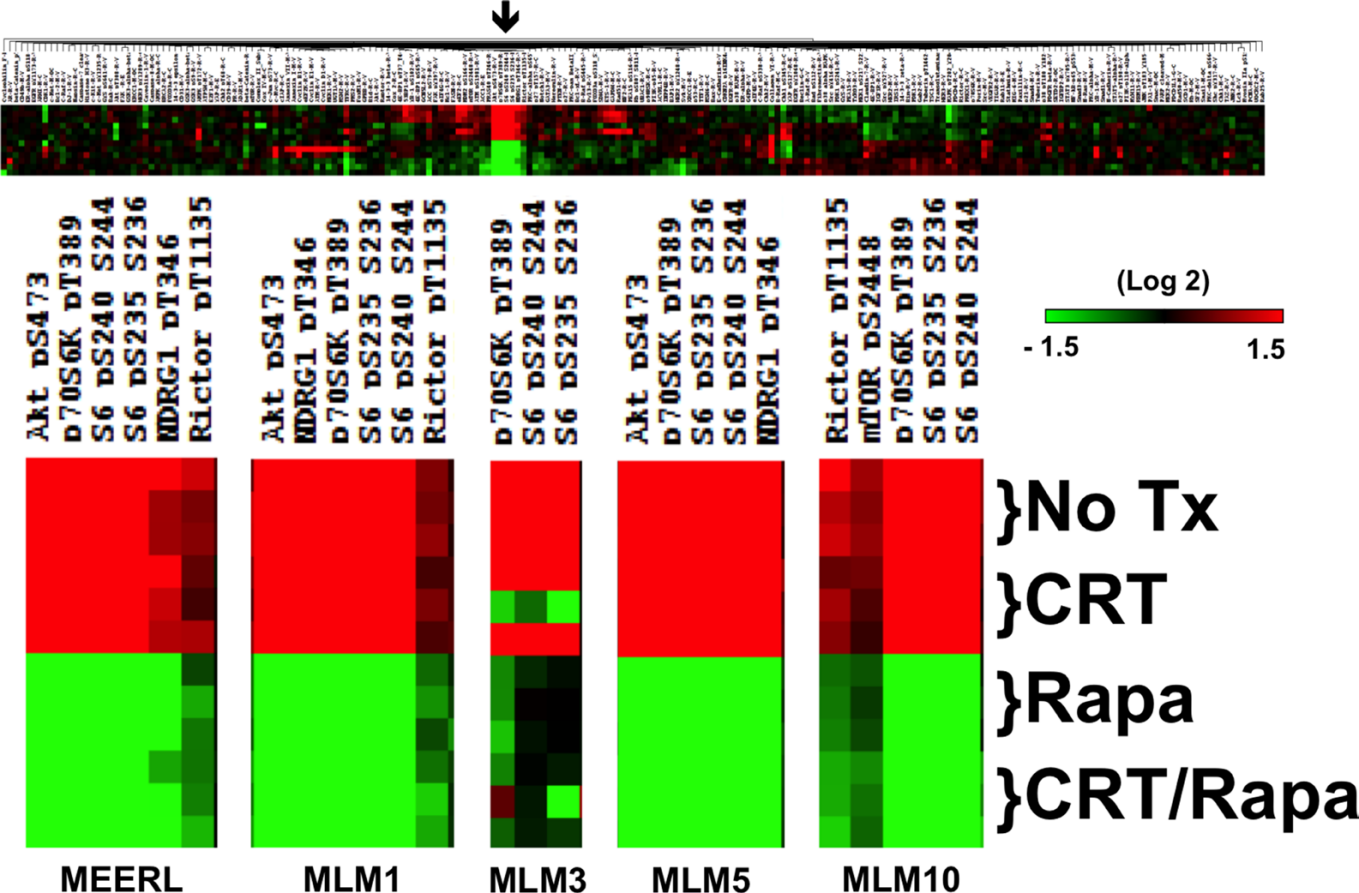
mTOR signaling in HPV+ OPSCC recurrent/metastatic disease RPPA results highlighting that mTOR activation is present in parental mEERL and all recurrent/metastatic MLM cell lines, as indicated by the fact that clusters of the highest levels of proteins (red) are activated, phosphorylated mTOR signaling proteins and downstream targets. Importantly, mTOR signaling is inhibited in all lines by rapamycin alone and in combination with CRT (shift to green). The full array for mEERL is shown as an example. The arrow indicates the region enlarged for each cell line individually below, showing changes under the indicated treatment conditions. Full-length arrays for each cell line can be found in Supplementary Figure 1.

Intra-tumor heterogeneity contributes to tumor evolution, progression, metastasis, and resistance to chemotherapy and radiation in head and neck cancer [14]. RPPA analysis was performed to define active signaling pathways and treatment-induced changes in mEERL and MLMs. Whole-cell lysates were harvested 24 hours after 1) no treatment, 2) CRT, 3) rapamycin, or 4) CRT and rapamycin (CRT/rapamycin). Protein expression was significantly different across cell lines (Supplementary Figure 1); however, mTOR signaling was consistently activated in each (Figure 1). mTOR signaling proteins demonstrating activation (red color) included P-Rictor (rapamycin insensitive companion of mTOR, a component of the mTORC2 complex), P-Akt s473 (mTORC2 phosphorylation site on Akt), P-S6K and P-S6 (direct downstream targets of mTOR), and P-NDRG1 (N-myc downstream regulated gene 1, a downstream target of mTOR induced by radiation and a key determinant in resistance to alkylating chemotherapeutic agents [15]). The full-length array for mEERL is shown as an example in Figure 1 alongside enlargements of these protein clusters for each cell line. Full-length arrays are included in Supplementary Figure 1. These results suggest mTOR signaling may be implicated in this murine model of HPV+ OPSCC and its recurrent/metastatic derivatives. Importantly, rapamycin inhibits activated mTOR-related phospho-proteins in all cell lines even in the context of CRT (Figure 1, green color). These data support emerging evidence that mTOR activation contributes to HPV+ OPSCC [13, 16], and correlates increased mTOR signaling with recurrent/metastatic disease. These data also suggest that mTOR remains targetable in resistant, recurrent/metastatic cells and that its inhibition is a feasible adjuvant to CRT.

### mTOR and metabolic activity are comparable or elevated in recurrent/metastatic HPV+ OPSCC cell lines

**Figure 2:**
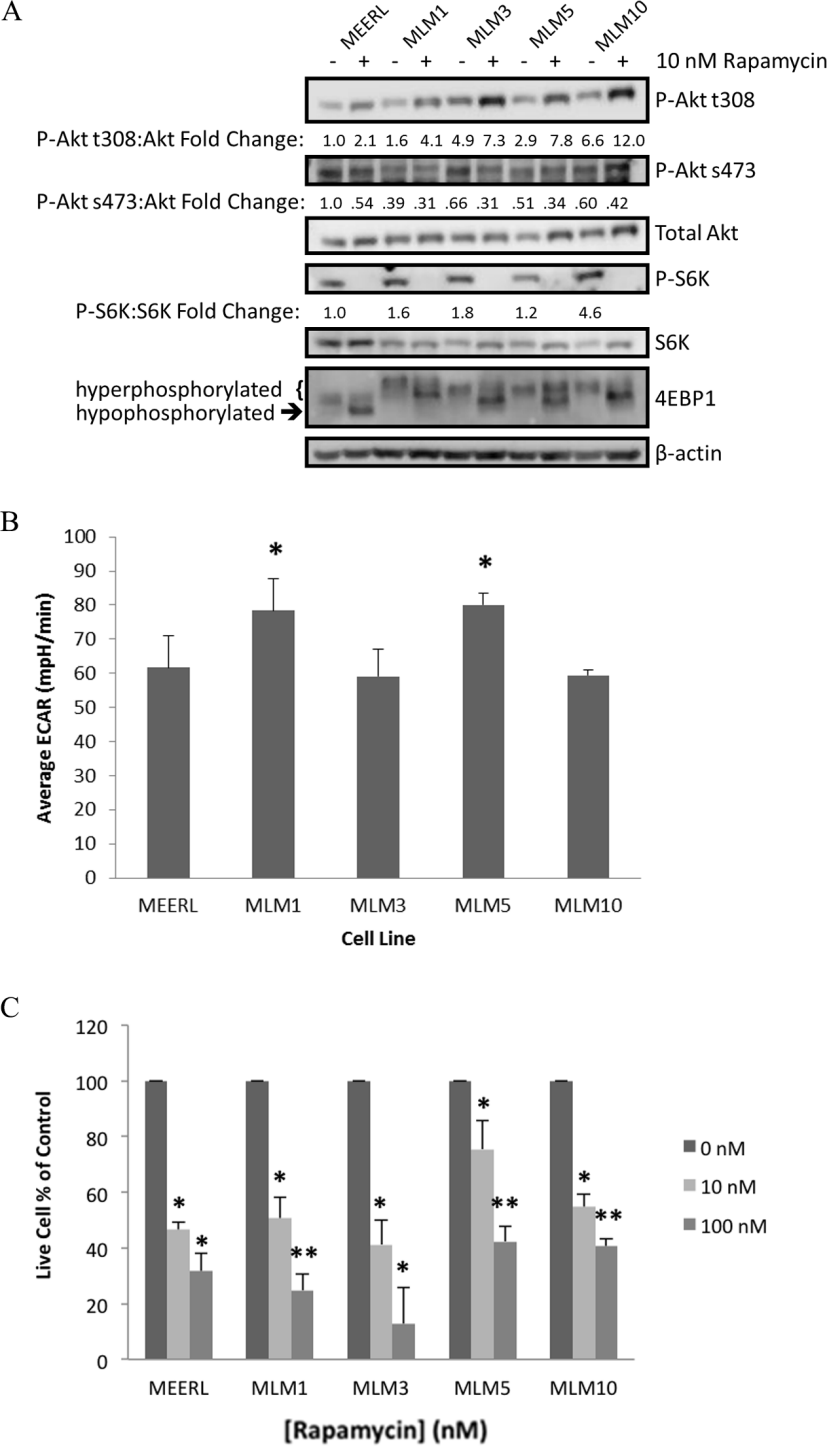
mTOR and metabolic activity are comparable or elevated in recurrent/metastatic HPV+ OPSCC cell lines (**A**) Western blot analysis of the mTOR pathway in each of the MLM cell lines and parental mEERL with and without rapamycin treatment. (**B**) Comparison of metabolic levels of each MLM cell line compared to parental mEERL, measured as ECAR by Seahorse. All MLM cell lines have metabolic levels comparable to mEERL (MLM3 and MLM10 ns) or elevated (MLM1 and MLM5 *p < 0.04). (**C**) Cell proliferation assays showing a dose response to rapamycin treatment in all lines and significant inhibition of cell growth even at low-dose (10 nM) rapamycin (*p ≤ 0.05 to control; **p ≤ 0.05 to control and **p ≤ 0.021 to 10 nM). SD is shown by error bars.

To validate the RPPA findings and determine the degree of mTOR activation and biochemical response to its inhibition, mEERL and MLMs were analyzed by western blot (Figure 2A). Whole-cell lysates were harvested 24 hours after treatment with 10 nM rapamycin or vehicle. Upstream of mTOR, basal levels of phospho-Akt (P-Akt) at the PI3K phosphorylation site (t308) were elevated in MLMs compared to mEERL. Accordingly, downstream P-S6K levels were also slightly elevated. Most strikingly, downstream phosphorylated 4EBP1 levels were likewise elevated in MLMs versus mEERL, indicated by higher levels of hyperphosphorylation. These data suggest that MLMs possess enhanced mTOR signaling compared to their parental mEERL cell line.

Loss of P-S6K and the shift to hypophosphorylated 4EBP1 indicate rapamycin-mediated inhibition of mTOR complex 1 (mTORC1), the central node of this pathway classically referred to as mTOR, in all cell lines. With rapamycin, compensatory increases in P-Akt t308 were also evident, but importantly, levels of P-Akt s473 decreased. These data suggest inhibition of mTOR complex 2 (mTORC2), consistent with previous reports [16]. mTORC2 is classically described as rapamycin insensitive, with mTORC1 defined by its rapamycin sensitivity. These data, however, suggest rapamycin mediates inhibition of mTORC1 and mTORC2 in all cell lines tested.

The mTOR pathway is a significant regulator of cellular metabolism, which we recently demonstrated and therapeutically exploited through mTOR inhibition in the parental mEERL cell line. We showed that rapamycin effectively attenuates cellular metabolism, linked to improved immune-mediated killing of the antigenic HPV+ cancer cells [13]. To determine whether MLM clones maintained the enhanced metabolic activity of their parental line, extracellular acidification rate (ECAR) was compared using the Seahorse XF24 system. MLMs had similar (MLM3 & MLM10 not significant (ns)) or elevated (MLM1 & MLM5 *p* < 0.04) metabolic activity compared to parental mEERL (Figure 2B), suggesting the anti-metabolic effects of mTOR inhibition may plausibly be substantiated to the recurrent/metastatic cells and providing further rationale for the use mTOR inhibition as a therapeutic strategy.

We next compared rapamycin sensitivity and its effect on proliferation by treating mEERL and MLMs with or without low- (10 nM) or high-dose (100 nM) rapamycin. Rapamycin significantly inhibited proliferation of all cell lines at 10–100 nM (*p* ≤ 0.05, Figure 2C). These data are consistent with RPPA analyses, which together suggest mTOR is both active and targetable in these recurrent/metastatic murine HPV+ OPSCC cells.

### Rapamycin enhances effects of cisplatin & radiation, sensitizing recurrent/metastatic cells to treatment

**Figure 3:**
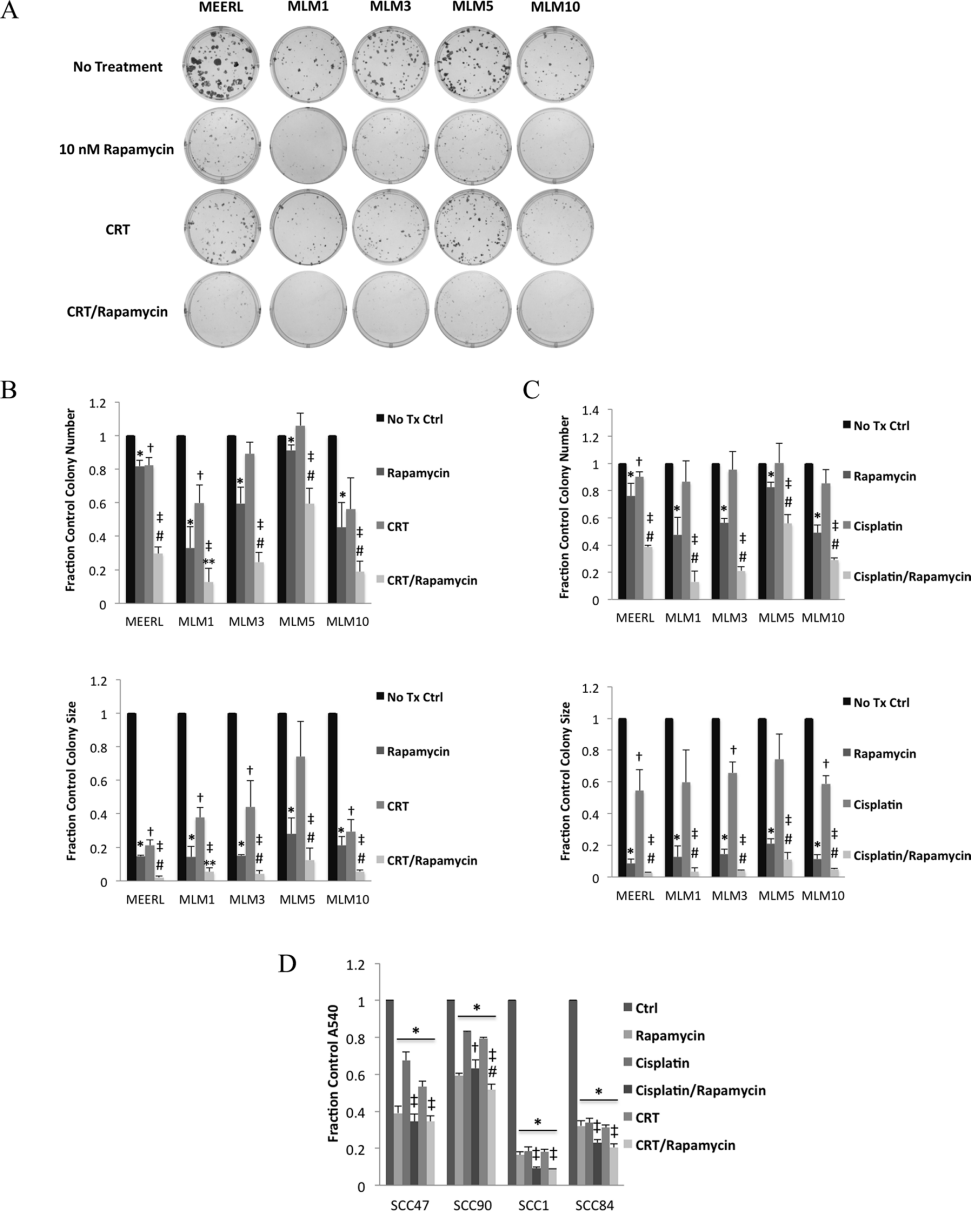
Rapamycin enhances effects of cisplatin & radiation, sensitizing recurrent/metastatic cells to treatment (**A**) Representative images of colonies of mEERL and each MLM cell line formed with no treatment, CRT, rapamycin, or their combination in clonogenic assays. Representative images for MLM1 and MLM10 can be found enlarged to show detail in Supplementary Figure 2. (**B**) Average colony number (Top; *p < 0.044 to control, ^†^*p* < 0.03 to control, ^‡^*p* < 0.02 to control, ^#^*p* < 0.02 to individual treatments, **p < 0.02 to CRT only) and size (Bottom; *p < 0.006 to control, ^†^*p* < 0.03 to control, ^‡^*p* < 0.003 to control, ^#^*p* < 0.05 to individual treatments, **p < 0.05 to CRT only) from the triplicate wells represented in A as a fraction of control. (**C**) Average number (Top; *p < 0.047 to control, ^†^*p* < 0.044 to control, ^‡^*p* < 0.008 to control, ^#^*p* ≤ 0.05 to individual treatments) and size (Bottom; *p < 0.003 to control, ^†^*p* < 0.03 to control, ^‡^*p* < 0.0009 to control, ^#^*p* ≤ 0.05 to individual treatments) of colonies of mEERL and each MLM cell line formed in clonogenic assays as above but instead using cisplatin alone, without radiation. (**D**) Crystal violet assays using two HPV+ (SCC47 & SCC90) and two HPV- (SCC1 and SCC84) human HNSCC cell lines. Absorbance of crystal violet destain solution, a surrogate of total cell number, is shown as a fraction of control for each cell line under each of the indicated treatment conditions (*p < 0.006 to control, ^‡^*p* < 0.04 to individual treatments, ^†^*p* < 0.04 to cisplatin only, ^#^*p* < 0.02 to cisplatin/rapamycin). SD is shown by error bars.

To test rapamycin adjuvantly to CRT, we analyzed the response of mEERL and MLMs to CRT, rapamycin, or their combination *in vitro* using clonogenic assays (Figure 3A). Decreases in colony number indicate cell killing, while decreases in colony size indicate growth inhibition. Rapamycin treatment alone significantly decreased number (*p* < 0.044) and size (*p* < 0.006) of colonies formed in all cell lines (Figure 3B). CRT significantly decreased size in all lines (*p* < 0.03) except MLM5, while significantly decreasing number of colonies in mEERL and MLM1 only (*p* < 0.03). These data suggest *in vitro* resistance to CRT in most of the recurrent/metastatic lines, particularly MLM5. However, combining rapamycin with CRT significantly decreased colony number (*p* < 0.02) and size (*p* < 0.003) in all lines. Moreover, CRT/rapamycin significantly decreased number (*p* < 0.02) and size (*p* < 0.05) of colonies compared to CRT or rapamycin alone in all lines except MLM1, where combined treatment was not significantly different from rapamycin alone in size or number. With the exception of MLM1 (most sensitive to mTOR inhibition *in vitro*), these data suggest rapamycin enhances CRT-induced cytotoxicity to HPV+ OPSCC cells, as previously described [13]. In addition, these data suggest rapamycin sensitizes recurrent/metastatic cells to standard treatment.

During treatment of distant metastases, metastatic sites are exposed only to systemically delivered agents, with radiation restricted as a local, primary therapy. Therefore, to determine if rapamycin could enhance the systemic toxicity of cisplatin, without radiation, we compared the cytotoxicity of these two agents individually and combined using clonogenic assays (Figure 3C). Rapamycin significantly decreased number (*p* < 0.047) and size (*p* < 0.003) of colonies in all cell lines. Cisplatin significantly decreased size in all lines (*p* < 0.03) except MLM1 and MLM5, but significantly decreased the number of colonies in mEERL only (*p* < 0.044), suggesting resistance to cisplatin in MLMs. Cisplatin/rapamycin, however, significantly decreased the number (*p* < 0.008) and size (*p* < 0.0009) of colonies in all cell lines. Furthermore, cisplatin/rapamycin significantly decreased colony number over that of either treatment individually (*p* ≤ 0.05). These data suggest that rapamycin enhances the cytotoxicity of the systemic agent, cisplatin, even in recurrent/metastatic cells.

To determine whether this effect also occurs in human cells, crystal violet assays were performed as above using two HPV+ (SCC47 & SCC90) and two HPV- (SCC1 & SCC84) human HNSCC cells lines. Crystal violet assays were employed over clonogenic assays (see Materials and Methods), as these cell lines do not form quantifiable colonies. All treatments significantly decreased absorbance (*p* < 0.006), indicating decreased cell number (Figure 3D). However, both CRT/rapamycin and cisplatin/rapamycin potentiated this decrease compared to individual treatments in all lines (*p* < 0.04) except SCC90, where cisplatin/rapamycin versus rapamycin was not significant. Interestingly, SCC90 is a known treatment-resistant cell line, derived from a tumor that failed therapy. This therapy resistance is evident compared to the other cell lines, however mTOR inhibition appeared to be the most effective denominator in treating these resistant cells *in vitro*. Radiation (per the CRT/rapamycin combination) did not significantly enhance the cisplatin/rapamycin effect except in SCC90 (*p* < 0.02), suggesting some degree of radio-sensitization unique to this therapy resistant line. Cisplatin/rapamycin being as effective as CRT/rapamycin in most human SCCs suggests, from a metastatic standpoint, the enhanced effects of this systemic combination may be sufficient to combat initiated or established metastases, and supports findings in the MLMs. Of course, *in vitro* experiments fail to take into consideration an essential component of combating HPV+ OPSCC, the immune response [13], which can only be assessed using an immune competent animal model.

### Rapamycin re-sensitizes recurrent/metastatic cell lines to treatment *in vivo*, significantly prolonging survival, improving long-term cures, and limiting lymph node and lung metastasis

**Figure 4:**
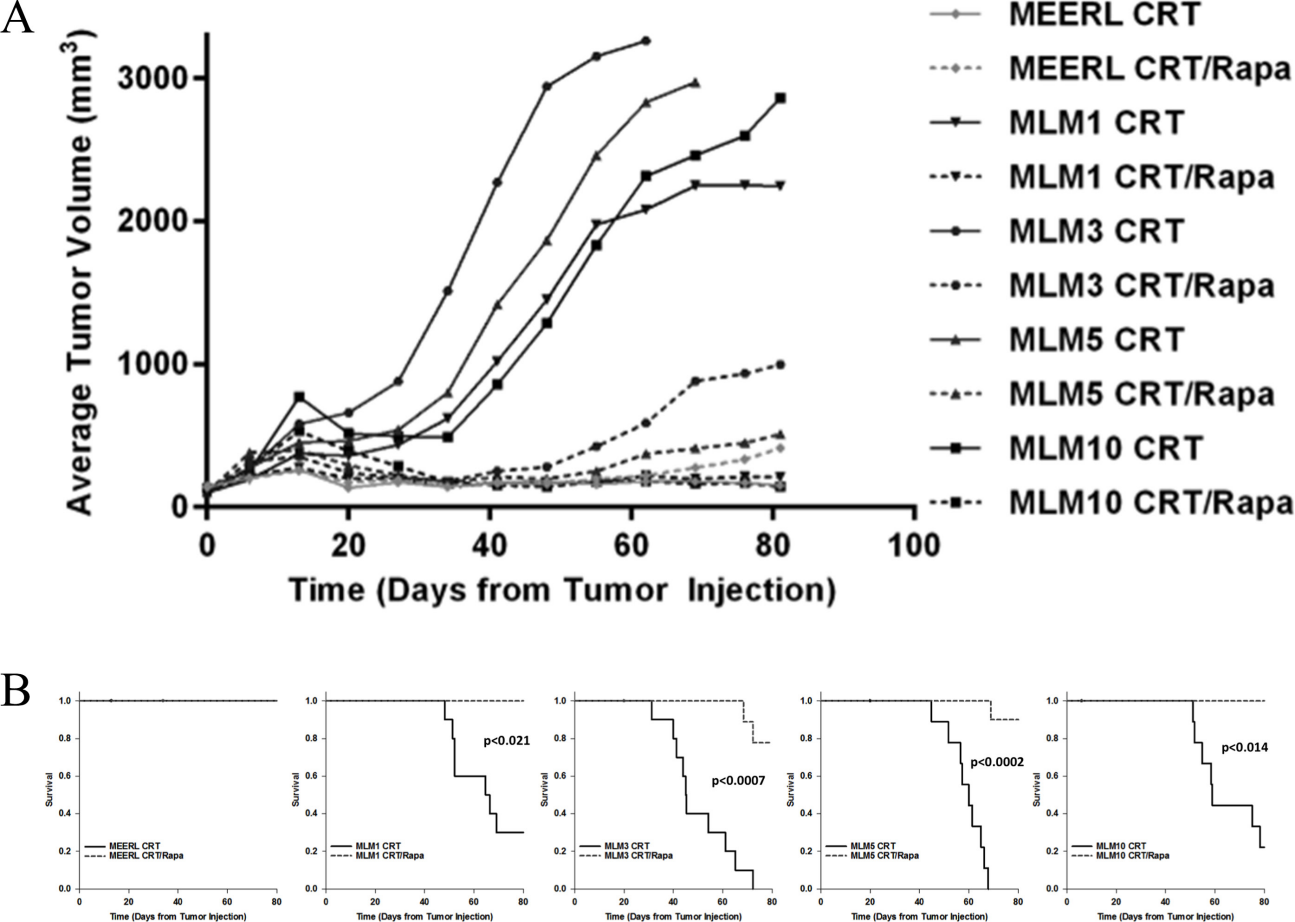
Rapamycin re-sensitizes recurrent/metastatic cell lines to treatment *in vivo*, significantly prolonging survival and improving long-term cures (**A**) Average tumor volume per cell line and treatment group. Solid lines represent tumor growth of CRT-treated mice, and dotted lines CRT/rapamycin treated mice. Both CRT and CRT/rapamycin treated mEERL tumors responded completely to treatment (ns to each other), while CRT-treated MLM tumors all showed significant resistance compared to mEERL (*p* < 0.021). The addition of rapamycin significantly re-sensitized all MLM tumors to treatment, evident in the separation of CRT to CRT/rapamycin treated MLM tumor growth curves (*p* < 0.001). *P*-values represent comparisons of average endpoint tumor volumes. *N* = 10 mice per group. (**B**) Survival plots for each tumor type comparing CRT to CRT/rapamycin treated mice. All CRT-treated mice with MLM tumors had significantly worse survival compared to mEERL tumor-bearing mice (*p* ≤ 0.04), indicating treatment resistance. However, the addition of rapamycin significantly improved survival with all MLM tumor types (*p* < 0.021; mEERL CRT to CRT/rapamycin ns).

Our data support the utility of the CRT/rapamycin combination in treatment of HPV+ OPSCC recurrent/metastatic disease. To test this, mEERL and MLM tumors were established (*N* = 20/cell line) and mice were segregated into two groups, receiving CRT alone or CRT and rapamycin. While both CRT and CRT/rapamycin treated mEERL tumors responded completely to treatment, CRT-treated MLM tumors all showed significant resistance compared to their parental line (*p* < 0.021). However, the addition of rapamycin re-sensitized all MLM tumors to treatment (*p* < 0.001) (Figure 4A); re-sensitization to standard CRT correlated with survival. While a complete response to both CRT and CRT/rapamycin was achieved in parental mEERL tumors, leading to 100% survival, MLM tumors were resistant to CRT and had significantly worse survival (0–30%; *p* ≤ 0.04, Figure 4B). Concurrent rapamycin, however, re-sensitized recurrent/metastatic cells to treatment, significantly improving survival in all MLM tumor types (78–100%; *p* < 0.021). These data correlate the mTOR pathway with resistance to CRT in these recurrent/metastatic HPV+ OPSCC tumors, and indicate that mTOR inhibition combines with CRT to improve therapeutic response.

**Figure 5:**
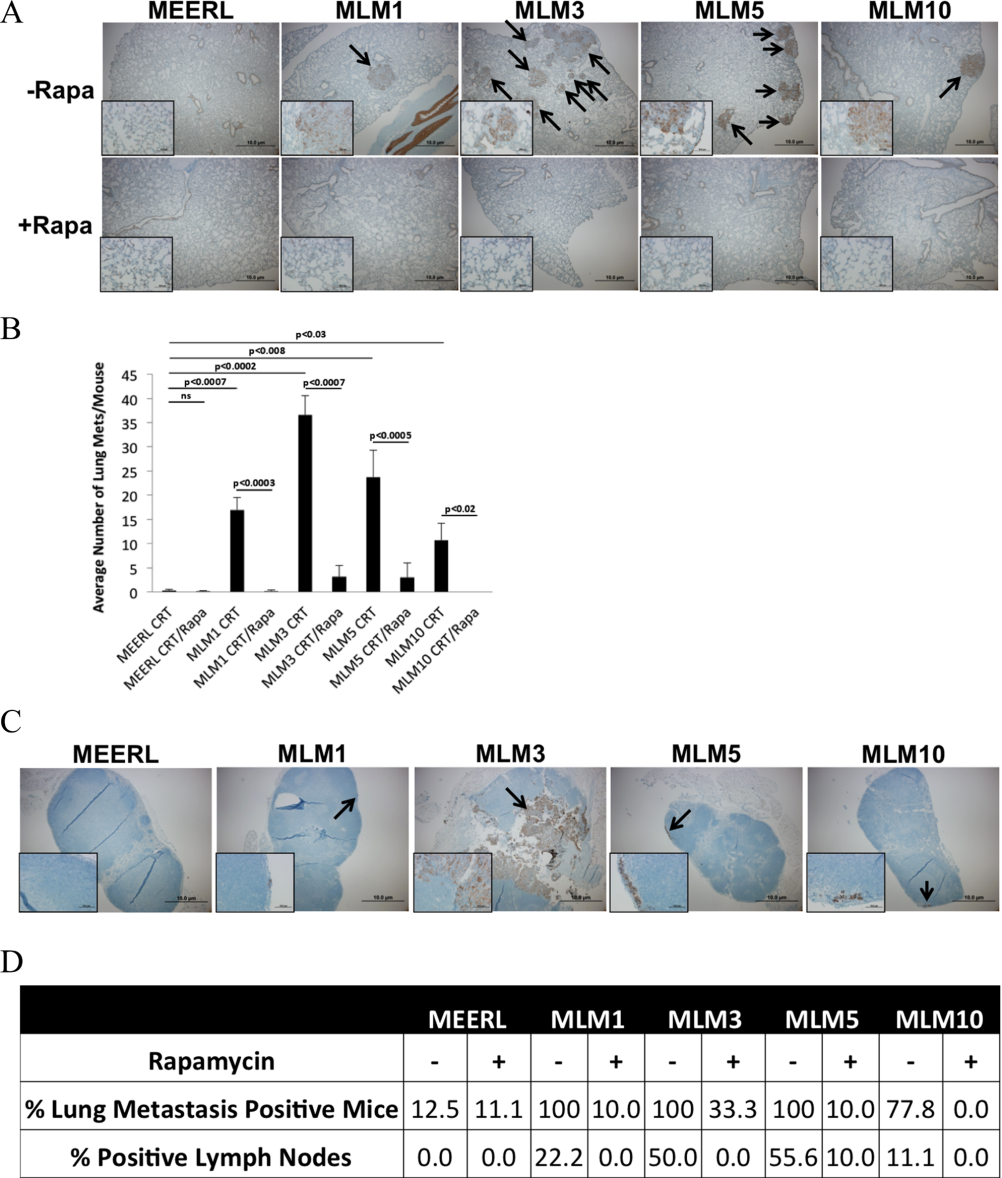
Adjuvant rapamycin limits lymph node and lung metastasis (**A**) Representative images of pan-cytokeratin staining (by IHC) of metastases in fixed, paraffin embedded lung tissue sections from each mouse treated with CRT (−Rapa) or CRT/rapamycin (+Rapa). Images were taken at 4x magnification, with insets at 40x to show staining. Arrows indicate metastases. (**B**) Correlating average number of lung metastases per mouse, averaged for each tumor type and treatment group. All MLM tumors gave rise to a significantly increased average number of lung metastases compared to parental mEERL with CRT alone (*p* < 0.03). CRT plus adjuvant rapamycin significantly reduced the number of lung metastases in all MLM groups (*p* < 0.02). SEM is shown by error bars. (**C**) Cytokeratin IHC showing an example of a positive draining inguinal lymph node of each MLM tumor type alongside a representative node from a mouse with a mEERL tumor. Images are at 4×, with insets at 40× to show staining. (**D**) Percent of lung metastasis positive mice and lymph nodes from each tumor type and treatment group.

While adjuvant rapamycin clearly improved therapeutic response of the recurrent/metastatic tumors, it remained unclear whether this response was restricted to the primary tumor or if it extended systemically. To quantify metastasis to the lungs and examine for tumor cells in the draining, inguinal lymph node, these tissues were harvested and analyzed by IHC for pan-cytokeratin. A significantly increased pulmonary metastatic burden was evident in all CRT-treated animals with MLM tumors compared to mEERL (*p* < 0.03); the addition of rapamycin significantly reduced this burden (*p* < 0.02) (Figure 5A and 5B). Rapamycin did not only limit metastatic burden at the lungs, but actually decreased the percent of mice with lung metastasis (Figure 5D). Though not all mice with lung metastasis had positive draining lymph nodes, the percent of positive lymph nodes also decreased with rapamycin treatment (Figure 5C and 5D). It is likely that inguinal lymph nodes were in the field of radiation, eradicating tumor in some animals. Alternatively, the FFPE sections may have missed the small number of tumor cells present in some nodes. Finally, it remains possible that the immune system cleared some nodal tumor cells. Regardless, the trend of decreased positive lymph nodes in rapamycin treated mice correlating with decreased lung metastatic burden suggests limited nodal dissemination.

Collectively, these data suggest that mTOR inhibition concurrent with standard CRT not only re-sensitizes resistant, recurrent/metastatic HPV+ OPSCC cells to treatment, leading to significantly prolonged survival and improved long-term cures, but that it also significantly limits lymph node and lung metastasis.

### Metastasis and the ability of rapamycin to limit it are independent of the adaptive immune response

**Figure 6:**
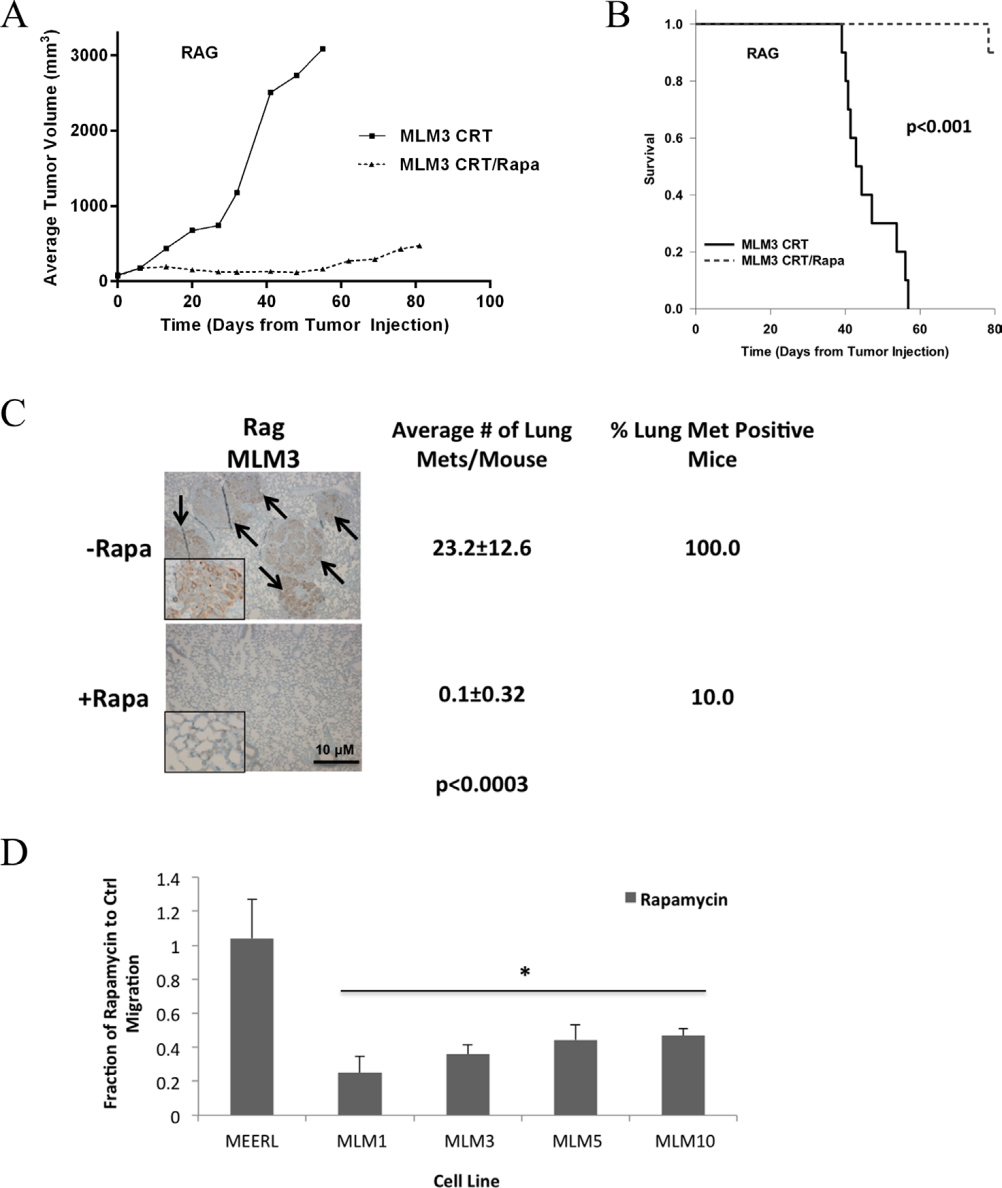
Metastasis and the ability of rapamycin to limit it are independent of the adaptive immune response (**A**) Average tumor growth and (**B**) survival data from MLM3 tumors treated with CRT and CRT/rapamycin in immunocompromised (Rag) mice. *N* = 10 mice per group. Nearly identical responses to those observed in wild-type mice resulted. Neither tumor growth nor survival were significantly different from wild-type mice under either treatment condition (Figure 4), and the addition of rapamycin still led to significantly inhibited tumor growth (*p* < 3.59 × 10^−5^) and improved survival (*p* < 0.001). Regarding tumor growth, *p*-values represent comparisons of average endpoint tumor volumes. (**C**) Representative images of correlating cytokeratin stained metastases in lung tissue alongside quantifications indicating a significantly reduced metastatic burden at the lungs and decreased number of metastasis positive mice with adjuvant rapamycin, as in wild-type mice. Images were taken at 4x magnification, with insets at 40x to show staining. Arrows indicate metastases. (**D**) *In vitro* migration assay as a basic measure of metastatic potential in the indicated cells treated +/− low-dose (10 nM) rapamycin. Rapamycin significantly limited migration of the metastatic MLM cells (*p < 0.05), but did not affect the migration of mEERL, which were very minimally migratory under either condition. SD is shown by error bars.

An adaptive anti-tumor immune response is induced by CRT and is necessary for clearance of HPV+ HNSCC [11]. We previously demonstrated that rapamycin enhances this immune response to parental mEERL tumors *in vivo*, improving clearance and long-term cures [13]. Immune and inflammatory responses are also integral to the metastatic process, modulating tumor growth-signaling, neovascularization, and tissue remodeling [17]. Therefore, we asked whether rapamycin similarly enhances the immune response to limit metastasis and whether an adaptive immune process is necessary to achieve metastasis. These questions were addressed by analyzing *in vivo* tumor growth and metastasis using one of the MLM lines and immune-compromised (Rag) mice. MLM3 was used in these experiments. MLM3 tumors were established and mice treated with CRT +/– rapamycin, with lungs harvested at endpoint. A nearly identical response of MLM3 tumors to CRT and CRT/rapamycin in wild-type was observed in Rag mice (Figure 6A, tumor growth; Figure 6B, survival). Furthermore, concurrent rapamycin significantly reduced pulmonary metastatic burden and decreased the number of metastasis positive mice (Figure 6C), as in wild-type mice. These data indicate rapamycin is not limiting metastasis by a mechanism requiring the adaptive immune response. In addition, the fact that lung metastases were still present in Rag mice indicates an adaptive immune process is also not required to achieve metastasis.

We then assessed whether rapamycin might limit metastasis more simply by interfering with a process such as cell migration. *In vitro* migration assays were used as a measure of metastatic potential +/– low-dose (10 nM) rapamycin. Rapamycin significantly limited migration of metastatic cells (*p* < 0.05), but did not affect that of mEERL, which were very minimally migratory under either condition (Figure 6D). With improved loco-regional control and enhanced systemic killing likely contributing, the data herein collectively suggest that rapamycin may limit metastasis through inhibition of mTOR regulated functions such as cell growth, proliferation, and motility, and may also include invasion, cytoskeletal rearrangement, and lymphangiogenesis, consistent with the published literature [3, 18].

## DISCUSSION

The mTOR signaling and downstream effector pathways are well described as implicated in oncogenesis, tumor progression, metastasis, and therapy resistance [18–23]. Our study utilizing four unique, clonal, lung metastatic OPSCC cell lines supports the role of mTOR signaling in these processes. This recurrent/metastatic model of HPV+ OPSCC closely recapitulates human disease in resistance to therapy, nodal dissemination, and lung metastasis. These cells show heterogeneity in protein expression and phenotype (see companion manuscript by Vermeer *et al.*), yet share activated mTOR signaling together with therapy resistance. Activation of mTOR can also occur in response to chemo- and radiotherapies, a described survival response as this potentiates key survival signals, which subsequently contributes to resistance [19, 20, 24]. mTOR activation providing growth and survival signals contributes not only to therapy resistance but also to progression and metastasis [18]. Importantly, we show that CRT with adjuvant rapamycin results in net mTOR inhibition, even in treatment-resistant, recurrent/metastatic cells. mTOR inhibition re-sensitized all MLMs to CRT and limited metastasis to regional lymph nodes and distantly to the lungs. Thus, these findings strongly correlate activated mTOR signaling with progression, metastasis, and therapy resistance in HPV+ OPSCC. mTOR is activated in more than 80% of head and neck cancers [25], underscoring its importance as a target for therapy.

The use of multiple targeting or dual-targeting agents (i.e. mTORC1/C2) to overcome feedback activation and resistance has garnered much interest in cancer research. The limitation of these approaches is increased toxicity, which renders many treatments and combinations intolerable. Of interest, the data presented here suggest rapamycin, considered mTORC1 specific unless in high doses and/or administered chronically [16, 21, 22], also inhibited mTORC2 in the tested cells. Feedback activation of mTORC1 through mTORC2's phosphorylation of Akt at serine residue 473 is often considered to be a significant limitation of an mTORC1 specific inhibitor [26]. However, inhibition of phosphorylation of both Akt s473 and targets downstream of mTORC1 were observed, even with chemoradiation and its potential activation of mTOR survival signaling [24]. This response may be unique to HPV+ OPSCC, perhaps tissue [16] or virus specific, allowing for inhibition of both mTOR complexes with a single complex targeting agent and the associated minimization of toxicities.

Taken together with our previous work showing improved clearance of primary HPV+ OPSCC with the addition of rapamycin to standard CRT [13], the findings presented here suggest the potential to limit recurrent/metastatic disease as a primary therapy. With its simultaneous inhibition of metabolic, growth, survival, and other tumor-promoting pathways, rapamycin may elicit a multifactorial antitumor response enhancing primary tumor clearance, re-sensitizing emerging resistant tumor cells to treatment, and limiting/preventing metastasis. Already FDA approved for human use, including numerous rapalogs with improved pharmacokinetic profiles [22], adjuvant use of mTOR inhibitors may be an ideal strategy to provide a well-tolerated treatment regimen that controls both loco-regional and recurrent/metastatic disease with translation to the clinic having the potential to be rapid. Though warranted in HNSCC in general, careful patient selection or retrospective analysis may be necessary, as HPV+, mTOR activated, or therapy resistant recurrent/metastatic disease may be of greatest benefit. Fortunately, advances in modern genomics, functional proteomics, and the availability of many therapeutic biomarkers along the PI3K/Akt/mTOR axis add to the promise of the proposed regimen not only in the potential to monitor treatment response but also in identifying those patients most likely to benefit [27]. However, as demonstrated particularly by the Gutkind laboratory, molecular targeting of mTOR seems a promising approach in HNSCC broadly, as 80–90% of all HNSCCs show activation of the PI3K/Akt/mTOR axis [19]. While 30% show genomic alterations in this axis, numerous other factors can lead to downstream mTOR activation, making exclusion of any HNSCC patients from future clinical trials involving mTOR inhibitors potentially premature [28]. Thus, future work evaluating the utility of the CRT/rapamycin combination in HPV-cancers, for which the proposed treatment appeared promising *in vitro*, is also warranted. Furthermore, establishing a cohort of samples from patients with known recurrent and metastatic disease to survey mTOR activation would help to identify a population of potential benefit.

It is worth noting that although the CRT/rapamycin combination ultimately limited lymph node and lung metastasis, it cannot be definitively stated that the mechanism was prevention. The possibility remains that enhanced systemic killing, demonstrated feasible in clonogenic assays, resulted in clearance of metastasizing tumor cells or established metastases. However, rapamycin treatment improved loco-regional control, decreased tumor cell positive lymph nodes, and limited migration of recurrent/metastatic cells, suggesting rapamycin preventing metastasis is likely. This is consistent with the literature, demonstrating inhibition of processes contributing to distant metastasis by rapamycin in HNSCC, such as lymph node metastasis and lymphangiogenesis [3].

While our previous studies demonstrate that mTOR inhibition may enhance direct CRT-induced cytotoxicity as well as the tumor-clearing immune response to primary HPV+ OPSCC as an adjuvant therapy [13], the work presented here substantiates use of this treatment combination to a lung metastasis model closely recapitulating human disease. Adjuvant mTOR inhibition may not only enhance treatment of the primary tumor, but re-sensitize emerging resistant, metastatic cell populations to treatment and limit lymph node and distant lung metastasis, improving long-term survival. The addition of an already FDA approved and well-tolerated mTOR inhibitor, such as rapamycin, to standard-of-care CRT may also provide a treatment option for patients with recurrent/metastatic disease, for which effective treatment options are currently lacking.

## MATERIALS AND METHODS

### Cell lines, culture conditions, and authentication

MOEs were previously derived from C57Bl/6 murine oropharyngeal epithelial cells [10]. The HPV+ OPSCC model cell line, mEERL [29], is routinely grafted into syngeneic mice for animal studies. MLM cell lines: MLM1, MLM3, MLM5, and MLM10, are characterized in the companion manuscript by Vermeer *et al*. Human **s**quamous **c**ell **c**arcinoma cell lines (**SCC**s) SCC1, SCC47, and SCC84 were originally generated at the University of Michigan (UM-SCCs) and received from Dr. Douglas Trask (University of Iowa) [30]. The SCC90 cell line was received from Dr. Randall Kimple (University of Wisconsin, Madison). The SCCs used were authenticated by Genetica DNA Laboratories via DNA profiling and routinely screened by our laboratory for HPV-16 mRNA (SCC47 and SCC90 are HPV+). SCCs were maintained in Dulbecco's modified eagle medium (DMEM, Hyclone) supplemented with 10% fetal bovine serum (FBS, Atlanta Biologicals), 100 U/mL penicillin (Hyclone), 100 μg/mL streptomycin (Hyclone), and 250 ng/mL amphotericin B (Cellgro). MOEs were maintained in DMEM supplemented with 22.5% Ham's F12 nutrient mixture, 10% FBS, 100 U/mL penicillin, 100 μg/mL streptomycin, 0.5 μg/mL hydrocortisone, 0.0084 μg/mL cholera toxin, 5 μg/mL transferrin, 5 μg/mL insulin, 0.00136 μg/mL tri-iodo-thyronine, and 5 μg/mL EGF (Invitrogen), referred to as E-media. Cell lines were maintained at a humidified 37°C in 5% CO_2_ and screened to ensure that they were free of mycoplasma.

### Extracellular acidification rate

Extracellular acidification rate (ECAR), a measure of metabolic rate, was measured using a Seahorse XF24 analyzer per manufacturer's protocol (Seahorse Bioscience). mEERL & MLMs were plated at 40,000 cells/well in XF24-well plates. The next day, cells were washed, equilibrated with non-buffered media for 30 min at 37°C in a CO_2_-free incubator, and transferred to the XF24 analyzer. Baseline ECAR was established followed by glucose injection to a final concentration of 10 mM. ECAR was measured every 5 minutes (3 minute mix, 2 minute wait) for 3 minute intervals. Time t_4_ (~16 mins) after glucose injection, a point when ECAR had begun to rise in all cell lines but before plateauing due to depletion of glucose, was arbitrarily selected to compare average ECAR of three replicates of each cell line.

### Cell proliferation assays

Cell lines were plated in triplicate and allowed to establish for 24 hours before treating with the indicated concentrations of rapamycin (LC Laboratories) or equal volume of vehicle (DMSO) in culture media. After 72 hours all cells were isolated, resuspended, and stained 1:1 with 0.4% trypan blue (Sigma-Aldrich). Live and dead cell numbers were counted via hemocytometer.

### Western blotting

Cell lysates were prepared and immunoblots performed as described previously [13]. Antibodies used: Phospho-Akt (Thr308, #4056, Cell Signaling), Phospho-Akt (Ser473, #05–669, Upstate), Akt (pan, #4691, Cell Signaling), Phospho-p70 S6 Kinase (Thr389, #9206, Cell Signaling), p70 S6 Kinase (#9202, Cell Signaling), and 4E-BP1 (R-113, sc-6936, Santa Cruz). Signal was detected using HRP-conjugated secondary antibodies and Amersham ECL prime detection reagent (GE healthcare). Exposures were captured using a CCD imaging system (MultiDoc-It UVP). Spot densitometry was performed using UVP software to quantify protein levels.

### Reverse phase protein array

Reverse phase protein microarray (RPPA) was performed by the MD Anderson Functional Proteomics Core Facility – (http://www.mdanderson.org/education-and-research/resources-for-professionals/scientific-resources/core-facilities-and-services/functional-proteomics-rppa-core/index.html). RPPA is a quantitative, antibody-based assay that determines levels of protein expression and modifications such as phosphorylation, cleavage, and fatty acid variation. RPPA allows concordant interrogation of multiple signaling molecules and their functional status. Each sample is analyzed for cell cycle progression, apoptosis, functional proteomics, and signaling network activity. We utilized RPPA to profile, compare, and validate signaling networks affected by CRT and rapamycin in MLMs. Heatmap generation and data analysis performed by MD Anderson. For more information, detailed methodologies, and the screened protein panel, see the above MD Anderson Core Facility website.

### Clonogenic & crystal violet assays

Cells were plated in triplicate at 367 MOEs/well on 6-well plates for each of the indicated treatments. The next day, cells were pre-treated with 10 nM rapamycin for 24 hours, mimicking our *in vivo* treatment regimen, before treatment with 0.5 μg/mL cisplatin (Calbiochem) dissolved in normal saline and radiation (4 Gy) with or without continued rapamycin. Cells receiving radiation were dosed one hour after this media change and returned to the incubator. Cisplatin concentration was determined from dose response data in previous work [11]. Four days later, cells were fixed with 70% ethanol and stained with 0.5% crystal violet (Fischer) in 10% ethanol. Average size and number of colonies (≥ 50 cells) were quantified using an AlphaImager system and corresponding software (AlphaInnotech). SCCs, which do not form quantifiable colonies, were plated at 10,000 cells/well (except SCC90 at 500,000 cells/well as they require confluency for growth) and thereafter treated as above. After fixing and staining, cells were washed with PBS, destained with 25% glacial acetic acid, and 100 μL of each sample's destain transferred to a 96-well plate with absorbance read at 540 nm as a relative surrogate of cell number.

### Migration assay

25,000 mEERL or MLMs/well were plated on 8-micron pore membrane migration chambers in 24-well format (BD Biosciences). Cells were plated in upper chambers in serum free media +/– 10 nM rapamycin; lower chambers contained complete media (10% serum). After 22 hours, chambers were aspirated, upper-membranes scrubbed, and cells that had migrated to the underside of the membrane fixed and stained with 70% ethanol and 0.5% crystal violet in 10% ethanol. Number of migrated cells from at least three representative images (20×) were averaged per membrane and counted manually.

### Animal studies

The *in vivo* model of HPV+ OPSCC was utilized as described previously [11], with the addition of MLMs. All experiments were performed in accordance with institutional and national guidelines and were approved by the Institutional Animal Care and Use Committee at Sanford Research. Briefly, 5 × 10^4^ mEERL or MLMs were injected subcutaneously (day 0) into the flank of syngeneic male C57Bl/6 (immune-competent) or B6.129S7-*Rag1*^tm1Mom^/J (RAG-1 or Rag) mice (immune-compromised, lacking mature B and T cells) (Jackson Laboratories). Mice weighed 20–25 grams. Tumors established for four days before initiating 21 days of 5 mg/kg intraperitoneal (IP) rapamycin or vehicle (5.2% Tween 80 (Sigma-Aldrich), 5.2% PEG-400 (Hampton Research) PBS [31]). Cisplatin/radiation therapy began two days later (day 6) and was given once a week for three weeks at 0.132 mg/mouse and 8 Gy (24 Gy total), respectively. Cisplatin dose is based on equivalent human dosing [11]. Due to their slower growth, mEERL and MLM10 tumors established for 8 days before treatment initiation. This timing eliminated likelihood of complete tumor clearance that would abrogate observance of any experimental therapeutic effect. Tumor dimensions were measured weekly using calipers to monitor growth. When all mice reached endpoint or a maximum of 80 days passed, Kaplan-Meier survival analysis was performed based on exponential regressions of individual tumor volume curves. Survival was standardized to the predefined 3000 mm^3^ tumor volume endpoint, as described previously [11]. Tumor growth curves represent average tumor volume of groups over time with maintained inclusion of endpoint volumes. Inguinal lymph nodes on the tumor side and lungs were harvested from each animal at endpoint, fixed in 10% buffered formalin, and paraffin embedded (FFPE).

### Tumor immunohistochemistry (IHC)

Anti-cytokeratin (1:100, ab9377, Abcam), an epithelial cell marker, was used for IHC analysis of paraffin embedded lungs and lymph nodes. Tissues were sectioned to 5 μm. A BenchMark^®^ XT automated slide staining system (Ventana Medical Systems, Inc) was used for staining with antibodies as per standard protocols. Slides were manually scanned and numbers of lung metastases per animal counted. The average number of lung metastases per tumor cell line and treatment group were calculated. Nodes were evaluated for the presence or absence of tumor cells.

### Statistical analysis

Error bars displayed are standard deviations from the mean of at least three replicates, unless otherwise indicated. Comparison of two groups was performed using two-tailed pairwise student's *t*-tests. Logrank significance tests were performed on Kaplan-Meier survival curves with *p*-values generated via the pairwise multiple comparisons Holm-Sidak method. RPPA data analysis was performed by MD Anderson (see method above). *p*-values ≤ 0.05 were considered significant.

## SUPPLEMENTARY MATERIALS FIGURES


