# Supplementary material for: mTOR inhibition as an adjuvant therapy in a metastatic model of HPV+ HNSCC
Source: Oncotarget. 2016 Mar 23;7(17):24228–41. doi: 10.18632/oncotarget.8286 (PMC5029697; doi:10.18632/oncotarget.8286)
Supplement: Supplementary file 1 [file oncotarget-07-24228-s001.pdf]

mTOR inhibition as an adjuvant therapy in a metastatic model of HPV+ HNSCC

Supplementary Materials

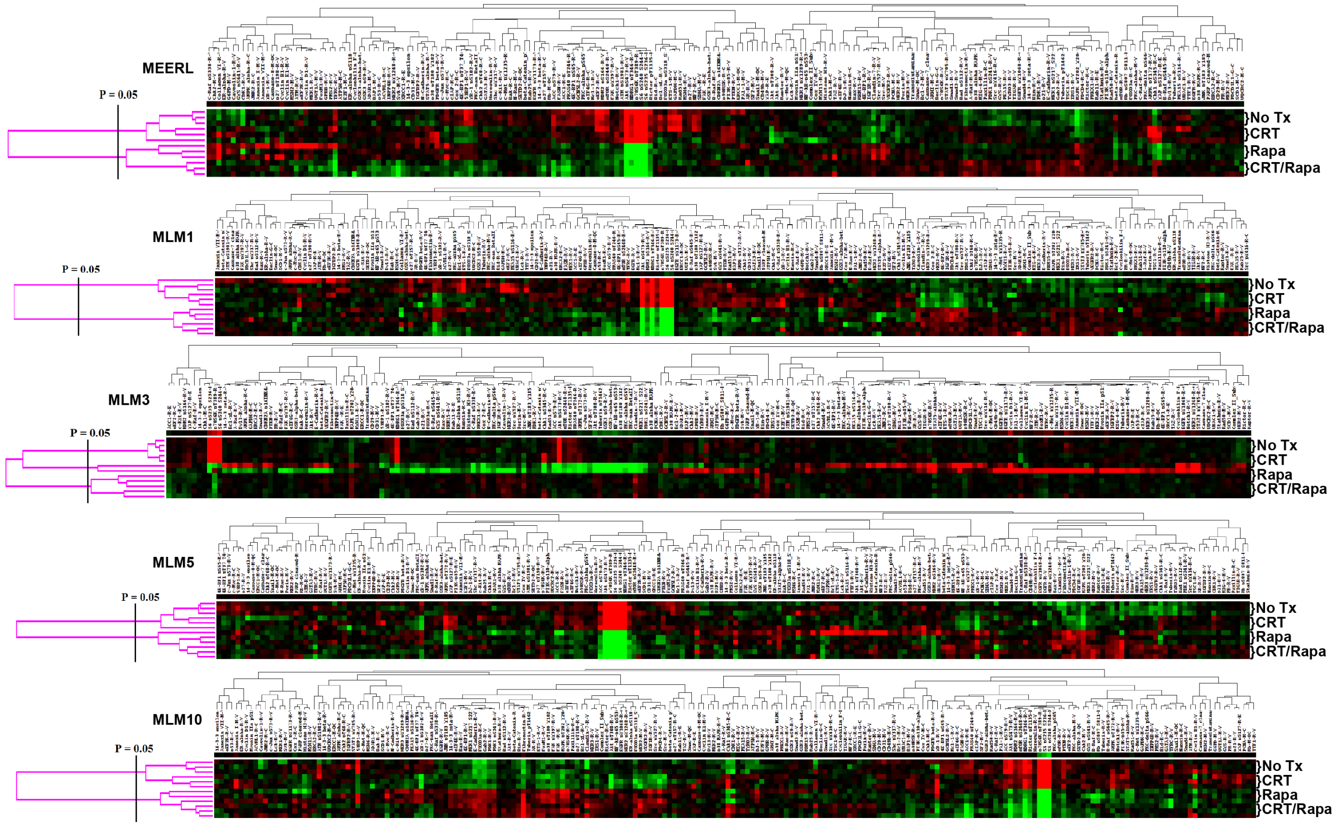

Supplementary Figure S1: Full-length reverse phase protein expression arrays with median centered unsupervised hierarchical clustering. Protein expression profiles of mEERL and each MLM cell line under conditions of no treatment (No Tx), CRT, rapamycin (Rapa), and the combination of CRT/rapamycin (CRT/Rapa) are shown, as detailed in the main text and corresponding to Figure 1. From top to bottom: mEERL, MLM1, MLM3, MLM5, & MLM10. Within each array, triplicates of no treatment, CRT, rapamycin, and CRT/rapamycin treated samples are arranged top to bottom, as indicated.

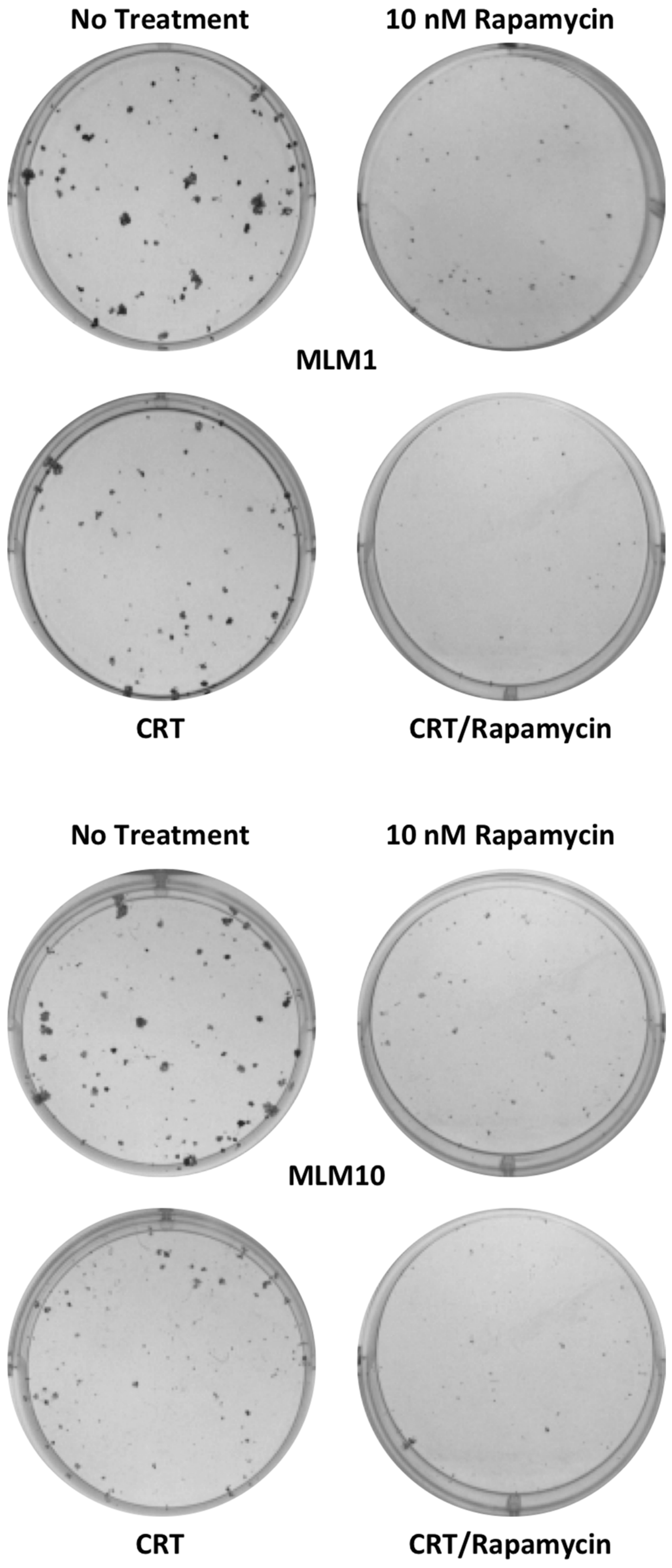

Supplementary Figure S2: Rapamycin enhances cisplatin & radiation induced cytotoxicity to OPSCC cells, sensitizing recurrent/metastatic cells to treatment. Representative images of MLM1 and MLM10 clonogenic assays enlarged from Figure 3 to show detail.
